# Transportation infrastructure and eco-environmental quality: Evidence from China’s high-speed rail

**DOI:** 10.1371/journal.pone.0290840

**Published:** 2023-08-29

**Authors:** Lan-ye Wei, Zhao Liu

**Affiliations:** 1 School of Business Administration, Chaohu University, Heifei, China; 2 Business School, Hunan University, Changsha, China; East China Normal University, CHINA

## Abstract

Ecological civilization construction is China’s national development strategy, and improving the urban eco-environmental quality is the key to accelerating this strategy, while the high-speed rail (HSR) opening is an important factor affecting the urban eco-environmental quality. Using panel data of 290 cities in China from 2004 to 2020, this study explores the impact of HSR opening on urban eco-environmental quality and its heterogeneity from the perspective of direct impact and interaction between HSR connected cities. Compared with cities without HSR service, the eco-environmental quality of cities with HSR service has significantly increased by 0.023 standard deviations, which is about 4.11% of the total change in urban eco-environmental quality in the same period. Second, there is an inverted U-shaped relationship between eco-environmental quality and urban space expansion. Third, the impact of HSR on eco-environmental quality is heterogeneous, mainly manifested in different cities and urban agglomerations. It means that the government should focus on the differences in the economic foundation and development characteristics of various regions, steadily push forward the construction and operation of the HSR, and speed up the renovation of existing lines to help the green development of cities. The research results provide a policy basis for the government to handle the relationship between infrastructure construction and eco-environmental quality, and effectively promote green sustainable development.

## 1. Introduction

The HSR, while playing the role of a transportation hub, has also reshaped China’s economic geography and affected the quality of the region’s ecological environment. China has built the world’s largest high-speed rail network, and the construction of new HSR cities and the development of new districts are booming, making China an ideal example for studying the environmental impacts of infrastructure development. On the one hand, as a fast, safe, convenient and environmentally friendly passenger transportation tool, the time-space compression effect brought about by HSR opening improves the flow and allocation efficiency of production factors in HSR-connected cities, strengthens spatial links between cities [1–3], and affects the quality of the ecological environment to a certain extent [4–6]. On the other hand, the construction of the HSR stations and the socio-economic activities arising from the HSR opening will accelerate the expansion of construction land and land use changes in the peripheral areas of the cities [7], resulting in habitat destruction, land resource development and ecological degradation [8]. In the context of the rapid development of China’s HSR network and the construction of a beautiful China, exploring the impact of HSR on urban eco-environmental quality and its heterogeneous characteristics is of great significance for coordinating the development and protection of land resources for HSR construction and realizing green development.

By the end of 2021, China’s HSR mileage in operation had reached 40,000 kilometres, and HSR had covered 93% of cities with populations of more than 500,000 people. Academic studies on the environmental impacts of HSR opening mainly focus on the assessment of the direct environmental effects brought about by the opening of HSR and the revelation of the mechanism of HSR opening on a single pollutant emission. Regarding the direct environmental impacts of the opening of HSR, scholars believe that the carbon emissions of HSR are low compared with other transportation modes [9, 10]; the role of the HSR opening on the emissions of different pollutants is heterogeneous [11]; and the role of the HSR opening on carbon emissions is uncertain from the perspective of the whole life cycle, considering the early construction of the HSR, the later maintenance, as well as the additional costs and resource wastage generated by replacing the ordinary railroads [12]. In terms of research on the role of HSR opening on pollutant emissions, the HSR opening helps to improve urban environmental efficiency and green productivity [13, 14], as well as curbing haze pollution [15]. In addition, direct land use resulting from the construction of the HSR, as well as land acquisition resulting from the production of raw industrial goods, could have environmental impacts [16]. During the important period of building a beautiful China, whether the opening of HSR can be used to realize the optimization of urban transport structure and resource integration to reduce environmental pollution, and whether it can produce a positive ecological and environmental effect by building sustainable transport deserves in-depth study. However, the research results on infrastructure construction and eco-environmental quality are relatively few and no consistent conclusions have been drawn. Moreover, the existing research on the mechanism of HSR affecting environment focuses on the characteristics of the city where the HSR station is located [17–19], and has not touched on the core network feature of transport infrastructure, that is, the differences between HSR connecting cities. This limits the understanding of ecological change during urbanization and suggests responses. In fact, identifying the factors affecting the eco-environmental quality and seeking effective paths to improve the eco-environmental quality are what the government tend to focus on, in formulating urban development policies and promoting high-quality economic development. Therefore, this paper will study the heterogeneity characteristics of urban transportation infrastructure affecting the eco-environmental quality by looking at the HSR network.

The marginal contributions of this paper include: First, it provides a new perspective for relevant literature. It is the enrichment and deepening of the research in this field to examine the impact, and heterogeneity of HSR construction, urban space expansion and eco-environmental quality in a unified analytical framework. Second, existing research focuses on the impact of HSR on a single environmental indicator [18], such as PM_2.5_. Moreover, compared with previous studies, this paper adopts the comprehensive evaluation method to measure the urban eco-environmental quality, to a certain extent, it avoids the poor representativeness caused by the selection of a single indicator, which makes the research variables more reasonable and scientific. This paper systematically examines the effect of HSR opening on eco-environmental quality. The difference in research perspectives enhances our understanding of the relationship between traffic layout and ecological environment, and can better guide local practice. Third, based on the interaction between HSR connected cities, this paper studies the heterogeneity of the impact of HSR opening on the eco-environmental quality to make up for the lack of existing research. This study is useful in accurately assessing the environmental benefits of the HSR network and providing a reference for environmental decision-making in the “high-speed rail era”. In the context of the global response to climate challenges and the urgent restoration of economic growth, the conclusions have important practical significance for developing countries to improve infrastructure and address environmental challenges.

Section 2 presents a literature review. Section 3 is the data and research methods. Section 4 discusses the empirical results. Section 5 introduces the heterogeneity analysis, and the final section is a summary and discussion.

## 2. Literature review

### 2.1 HSR cities versus non HSR cities

As a typical representative of the upgrading of traditional transportation infrastructure, HSR is different from other modes of transportation, which mainly focuses on passenger transportation. The most important role of HSR is to improve people’s travel efficiency through spatio-temporal compression effect, and HSR services are competitive in terms of network connectivity and cost efficiency [20]. The direct impact of the HSR opening on eco-environmental quality is represented in the following three aspects. First, compared with non-high-speed rail cities, the opening of HSR will generally bring about urban land expansion and promote land urbanization [21, 22]. Looking from the perspective of land use structure change, the construction of the HSR will affect the eco-environmental quality, for example, the HSR opening leads to the conversion of vegetated land and cropland to urban land [23]. The reason is that in most Chinese cities, HSR stations are newly built and mostly located in the suburbs. Many literatures have confirmed that China’s HSR construction leads to urban land expansion. Second, HSR construction improves the efficiency of the intercity transportation system, but also creates and transfers travel demand [24, 25]. Traffic pollution is an important factor affecting eco-environmental quality [26]. Finally, the impact of HSR on eco-environmental quality lies in its environmental externalities. On the one hand, it makes the market of production factors such as labor, resources and technology more accessible to enterprises, and expands the selection range of human capital, material capital and technology [27]. Based on the transaction cost theory, the HSR opening can reduce pollution emissions by improving the resources use efficiency [28, 29]. On the other hand, the spatio-temporal compression effect of the HSR can shorten the distance between producers and demanders, reduce the waste of resources caused by the mismatch between supply and demand, and reduce the opportunistic behavior caused by information asymmetry [30], thus helping to enhance eco-environmental quality [31, 32]. In conclusion, the impacts of HSR construction and operation on ecological and environmental quality are complex. When the urban land use efficiency is high and HSR can integrate traditional transportation mode and play a resonance effect, it is expected that the HSR can improve eco-environmental quality.

### 2.2. HSR network connects cities

From the theoretical level, the HSR can change the spatial distance between regions, so as to have a profound impact on economic growth and industrial development. The differences in population size [33], industrial structure and economic growth among cities within the HSR network are the root causes of the interaction of economic activities between cities [34, 35].

First, infrastructure upgrading plays an important part in affecting regional economic activities, mainly manifested as siphon effect and diffusion effect [36, 37], that is, the role of infrastructure in the economy has regional heterogeneity [38, 39]. In the early stage of economic growth, labor factors, technology and knowledge will converge to regions with better infrastructure, thus showing the “echo effect” (“siphon effect” or “polarization effect”). However, as the economy continues to develop and the further improvement of infrastructure, the elements of the central area will radiate to the surrounding areas, resulting in a “diffusion effect” [40, 41]. Second, the differences in economic development level, population size and industrial structure of cities connected by HSR will lead to the heterogeneity of economic exchanges between the city and other cities. For example, in the context of continuous strict ecological environmental governance, on the one hand, China’s developed cities have set a high threshold for environmental protection. Big cities make full use of their advantages in technology and human resources to focus on developing knowledge and technology intensive industries, such as finance and other high-end manufacturing. Then, the labor-intensive industries will be transferred to small and medium-sized cities connected with large cities through HSR with relatively low labor and land costs. On the other hand, large cities transfer their production departments to medium and small cities connected with HSR, and retain R&D, sales and other departments to facilitate business expansion, which will also lead to pollution transfer through HSR connectivity and interaction. In addition, small and medium-sized cities tend to take over polluting industries that have been transferred from large cities to create jobs and accelerate economic development. This means that cities in different development stages, population size and industrial structure have heterogeneous impacts on urban eco-environmental quality through the interaction of HSR network. Conversely, the operation of HSR will also influence the industrial structure through: division of labor, convergence effect, and learning effect among cities [42]. It can be seen from literature review that the role of transport infrastructure upgrading represented by HSR in economic development, industrial structure and labor mobility is controversial [43–45]. However, the common point that many scholars dispute is that HSR has heterogeneous impact on economic activities in big cities, small and medium-sized cities, developed regions and underdeveloped regions [46–48].

According to the theory of environmental economics, population is the most direct cause of human pressure on regional ecosystems [49–52]. Cross-regional labor flow is a normal state in China’s rapid urbanization process. Labor flow plays an important role in the process of HSR operation affecting eco-environmental quality [53]. There is no doubt that HSR reshapes the urban industrial structure [54], population structure and employment structure by affecting the flow of population to interconnected cities [43, 55, 56]. In particular, the large-scale migration of rural labor (within the jurisdiction of small or medium cities) to big cities, which will affect eco-environmental quality from all aspects. Different from other transportation modes, HSR is mainly for passenger transport. In this context, the HSR has facilitated the cross-regional movement of China’s large population. The statistics from China’s National Bureau of Statistics (NBS) show that China’s floating population was 280 million in 2019, accounting for 20% of the total population in the same period. The main reason is that China’s “pilot” development strategy implemented in some regions after the reform and opening has contributed to the rapid economic growth (e.g. Shenzhen, Xiamen), attracted many migrant laborers and made an important input to economic growth. The fundamental reason for the large-scale cross-regional mobility of Chinese labor force lies in the gap in the basis of economic development, which is specifically manifested as industrial structure and income level. Therefore, it is necessary to distinguish the industrial structure, population size and economic development level of cities connected by HSR and explore the impact of HSR opening on the eco-environmental quality. To a certain extent, it also helps to reduce the estimation bias induced by the urban selection effect of the individual dimension and the agglomeration effect of the urban dimension.

### 2.3. The summary

The above analysis shows that the construction process of the HSR station and the HSR operation will affect eco-environmental quality. Transportation infrastructure upgrading has a direct or indirect impact on eco-environmental quality, especially the trans-regional flow of labor and other factors has agglomeration effect or even siphon effect. In reality, due to the huge HSR network in China and the huge differences in economic development, industrial structure, population size and other aspects between cities, the impact of HSR connectivity between different node cities on the eco-environmental quality may be huge differences, which is the issue discussed in this empirical study.

## 3. Model construction and data source

### 3.1. Variable selection and indicator description

#### 3.1.1. Eco-environmental quality

Based on the “P-S-R” model, this paper constructs the eco-environmental quality system evaluation index system [57–59]. The detailed indicator system is shown in Table 1. The entropy method is used to objectively analyse the original information to determine the index weight and calculate the eco-environmental quality of each city [60]. To dissipate the role of indicator dimensions on evaluation results, in calculating the eco-environmental quality, the data of each evaluation index was first standardized by using the range method to determine the indicator weight. Finally, the information entropy of each index is counted by normalization, and the weight of each index is obtained.

**Table 1:** Construction of eco-environmental quality indicators.

**Eco-environmental quality (leeq)**	Pressure	CO_2_ emissions	Tons (-)
		Surface PM_2.5_	% (-)
		Surface SO_2_	Microgram per cubic meter (-)
	State	Normalized Difference Vegetation Index (NDVI) (monthly maximum)	% (+)
		Proportion of cultivated land and construction land	% (+)
		Days of inversion	% (-)
		Per capita water supply of the city	10000 m^3^/person (+)
	Response	Comprehensive utilization rate of industrial solid waste	% (+)
		Local governments’ attention to ecological environment	% (+)

#### 3.1.2. Control variables

The Control variables. (1) Urban space expansion (US). The ratio of urban built-up area to permanent population is used to measure urban space expansion. (2) Economic development level (lnpgdp). The per capita GDP of the city is used to represent the economic development level. (3) The industrial structure (INS) is calculated as a percentage of GDP from the value added of the secondary sector. (4) Human capital (HC). It is expressed by the proportion of students in urban ordinary institutions of higher learning to the population. (5) Technological innovation (RD), measured by the ratio of urban government R&D expenditure to fiscal expenditure. (6) The ratio of real FDI to GDP is used to express the openness of the economy (FDI) [61]. In this paper, the allocation of environmental protection attention of each city is mined based on Python, and the proportion of keyword frequency (including: environmental pollution, CO_2_, SO_2_, haze and other words) in the total word count of the report is calculated by referring to the research of relevant keywords in government texts. Based on the CO_2_ emissions generated by energy consumption at the production end, eight kinds of energy, eight energy sources such as raw coal, coke and crude oil are selected as final consumption, and carbon emissions are calculated according to the consumption [62]. Surface PM_2.5_, Surface SO_2_, and days of inversion are from the NASA’s M2TMNXAER_ 5.12.4. HSR related data, derived from CNRDS database (https://www.cnrds.com), include the construction and opening of HSR lines since 2003, as published by the State Railway Administration, and other data are obtained from the statistical yearbooks of Chinese cities, Wind database and EPS DATA database. Some missing data are supplemented by interpolation. Data involving value patterns are deflated using the relevant deflators, and the resulting ratios are logarithmically processed. Table A1 in S1 File reports descriptive statistics for the main variables.

### 3.2. Empirical strategy

To study the influence of HSR construction on the eco-environmental quality, it needs to compare the eco-environmental quality of cities before and after the opening of HSR. However, it is impossible to draw objective conclusions by direct comparison, because the difference in ecological quality before and after the opening of HSR may not be driven by the construction of HSR, but by other factors. The Multi-period DID method of constructing counterfactual groups is used to solve this problem in the paper. The model is as follows:

$$\ln leeq_{it}=\beta_{1}+\beta_{2}HSR_{it}+\beta_{3}X_{it}+\mu_{i}+\delta_{t}+\varepsilon_{it}$$ (1)

where, *i* and *t* represent city and time respectively, and *lnleeq* represents eco-environmental quality. *HSR*_*it*_ means that the HSR of city *i* is opened for the first time in year *t*. If the HSR is opened in year *t*, the value is 1, otherwise the value = 0. *β*_*2*_ is the net impact of HSR construction on eco-environmental quality. If *β*_*2*_>0, it means that HSR helps to improve eco-environmental quality. *X* is the set of control variables. *μ*_*i*_ is an individual fixed effect, which is used to control the impacts of time-independent or unobservable factors on eco-environmental quality, such as the city’s annual average temperature, the city’s elevation and terrain complexity, and the distance from the city to the nearest port. *δ*_*t*_ is a time fixed effect to control factors that may affect eco-environmental quality, such as macroeconomic conditions, national environmental policies, and macroindustrial policies. If the HSR of the city starts to operate from January to June, it will be considered to open in this year; If the HSR starts to operate from July to December, it will be deemed to be opened in the next year. If a city has opened multiple HSR lines, the previous year shall be used as the date of HSR opening in the city. In this paper, the Beijing-Tianjin Intercity Railway, which was opened to traffic in 2008, is regarded as China’s first HSR. In addition, to match what is frequently done in DID estimation, the standard errors are clustered at the city-level.

### 3.3. Empirical model-heterogeneity analysis

In this paper, cities are grouped according to the economic development level, industrial structure index and population size, and the panel data double fixed effect model is used to investigate the impact of HSR connection with different types of cities on the eco-environmental quality. The models are set as follows:

$$\ln leeq_{it}=\gamma_{10}+\gamma_{11}HSR_{it}+\gamma_{12}HSR_{-hpgdpit}+\gamma_{13}HSR_{-lpgdpit}+\lambda_{14}X_{it}+\rho_{i}+\theta_{t}+\upsilon_{s}t+\varepsilon_{it}$$ (2)

$$\ln leeq_{it}=\gamma_{20}+\gamma_{21}HSR_{it}+\gamma_{22}HSR_{-hisit}+\gamma_{23}HSR_{-lisit}+\lambda_{24}X_{it}+\rho_{i}+\theta_{t}+\upsilon_{s}t+\varepsilon_{it}$$ (3)

$$\ln leeq_{it}=\gamma_{30}+\gamma_{31}HSR_{it}+\gamma_{32}HSR_{-hpopit}+\gamma_{33}HSR_{-lpopit}+\lambda_{34}X_{it}+\rho_{i}+\theta_{t}+\upsilon_{s}t+\varepsilon_{it}$$ (4)

*HSR_*_*hpgdp*_ and *HSR_*_*lpgdp*_ reflect the connection with cities with different economic development levels via HSR respectively. *HSR_*_*hpgdp*_ = 1 indicates that city *i* is connected to cities with higher economic development level than itself through HSR in year *t*, and 0 indicates that there is no. On the contrary, *HSR_*_*lpgdp*_ means that city *i* is connected to cities with lower economic development level than itself by HSR in year *t*, and a value of 0 means that there is none. Two cities are considered “connected” as long as they are both stops of a HSR train (including the starting, intermediate and terminal stations), or if there is a direct HSR train between them. Specifically, we divide all urban samples into four groups based on the economic development level at the beginning of the year. If cities in the lower group are connected with cities in the higher group by HSR, then *HSR_*_*hpgdp*_ = 1 and *HSR_*_*lpgdp*_ = 0, and vice versa; if it is connected to the same group of cities, then *HSR_*_*hpgdp*_ = 0 and *HSR_*_*lpgdp*_ = 0. Similarly, in Eqs (3) and (4), we adopt the same processing method to define indicators *HSR_*_*his*_, *HSR_*_*lis*_, *HSR_*_*hpop*_ and *HSR_*_*lpop*_.

### 3.4. Common trend test

An important premise of the DID model is that the key variables of the experimental group and the control group before policy implementation should meet the common trend hypothesis. In other words, it was assumed that the difference of eco-environmental quality between the treatment group and the experimental group would not change significantly over time without the HSR in service. The dynamic DID model is used to test the equilibrium trend [63]. The model is as follows:

It is assumed that the difference in eco-environmental quality between the treatment group and the experimental group will not change significantly over time before the operation of HSR.


$$\ln leeq=\delta_{0}+\sum_{T\in \{-4,\cdots -2,-1,0,1,2,3,\cdots 10,11\}}\delta_{T}\times T_{it}\times HSR_{it}+\delta X_{it}+\mu_{i}+\delta_{t}+\varepsilon_{it}$$ (5)

**Fig 1:**
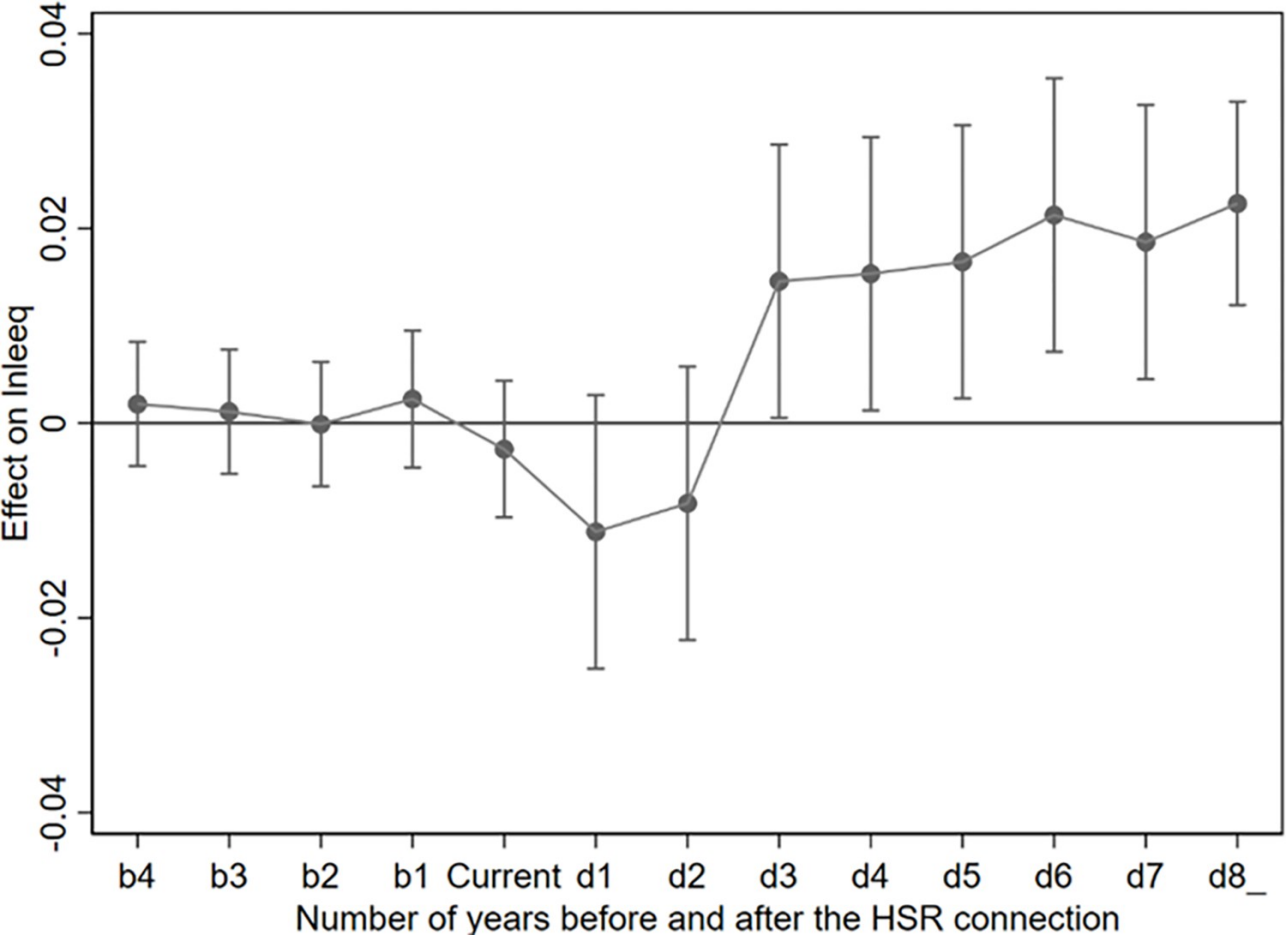
Results of parallel trend test.

At the 10% level, the regression coefficients of b1-b4 were not significant, and were close to 0 in Fig 1, indicating that there was no significant difference between the experimental group and the control group before the opening of HSR. It is worth noting that the estimated coefficients of Current, d1 and d2 are negative and not significant, which means that the first two years of HSR operation may have negative effects on urban eco-environmental quality. Therefore, the result of parallel trend test is valid. According to the estimated coefficients of d3-d8_, two years after the HSR was officially opened, the eco-environmental quality has been significantly improved.

## 4. Empirical results

### 4.1. Basic estimation results

The DID model was used to examine the effects of HSR construction on eco-environmental quality. The Hausman test showed statistically significant difference between the original coefficients representing fixed effects and random effects, strongly reject the null hypothesis. Hence, the estimation results of random effects are biased. Based on controlling for individual fixed effects and time fixed effects, a quadratic term of urban space expansion and economic growth was added to the model.

As shown in Table 2, the regression coefficient of HSR_it_ is significantly positive whether or not control variables are included, indicating that HSR construction has significantly improved eco-environmental quality. To better visualize the impact of HSR opening on the eco-environmental quality, we calculated the standard deviation change of eco-environmental quality according to the coefficient estimation result of HSR, and also calculated the ratio of the changes in the eco-environmental quality brought about by HSR opening to the total changes in eco-environmental quality. The results show that the HSR opening has significantly increased the eco-environmental quality by 0.023 standard deviations, which is about 4.11% of the total change in eco-environmental quality in the same period. It means that compared with the investment in railways, although the construction of HSR has a disadvantage at the ecological level at the initial stage [16], after the opening of HSR, the environmental protection and other features of HSR show the ecological advantage [64].

**Table 2:** Impact of HSR construction on the eco-environmental quality: fixed effect model.

Variable	(1)	(2)	(3)	(4)	(5)	(6)	(7)
**HSR**_**it**_	0.068** (0.027)	0.028*** (0.008)	0.023*** (0.007)				
**lnfrequencies**_**it**_				0.123*** (0.004)	0.004*** (0.001)		
**Routes**_**it**_						0.046** (0.021)	0.027*** (0.010)
**US**		3.798*** (0.574)	2.624*** (0.463)		1.096*** (0.340)		2.589*** (0.463)
**US** ^**2**^		-1.514*** (0.248)	-1.329*** (0.197)		-0.459*** (0.147)		-1.325*** (0.197)
**lnpgdp**		-6.243*** (0.435)	-6.216*** (0.438)		-4.857*** (0.451)		-6.199*** (0.463)
**sqlnpgdp**		0.280*** (0.021)	0.277*** (0.021)		0.214*** (0.021)		0.276*** (0.022)
**HC**			0.029*** (0.011)		0.013*** (0.004)		0.027*** (0.011)
**FDI**			-0.037 (0.164)		-0.069 (0.150)		-0.032* (0.167)
**INS**			-0.069 (0.150)		-0.407* (0.217)		-0.933*** (0.353)
**RD**			0.024** (0.009)		0.012** (0.006)		0.025** (0.010)
**N**	4,760	4,663	4,259	2,808	2,730	4,660	4,259
**Adj -R** ^**2**^	0.776	0.963	0.969	0.855	0.980	0.771	0.969

Note

Inside the bracket is the standard error of cluster and city

*p<0.10

**p<0.05

***p<0.01.

In addition, the frequency of HSR trips and the number of HSR lines were used as proxies for the variable of whether HSR was opened to test the marginal impact of HSR construction on eco-environmental quality. The results are displayed in columns (4)—(7). It shows that every 1% increase in travel frequency will improve the eco-environmental quality by about 0.4%, and the eco-environmental quality will be improved by about 1.2% for each additional HSR station. This is supported by Sun, Yan [33], who found that HSR significantly improves the environmental efficiency of Chinese cities. Second, the relationship between economic growth and the eco-environmental quality conforms to the environmental Kuznets curve [65], and Chinese cities have not yet reached the inflection point. Third, the results in Table 2 also show that the relationship between urban space expansion and the eco-environmental quality is U-shaped, that is, appropriate space expansion is conducive to improving eco-environmental quality, but beyond the inflection point, excessive spread will damage the urban eco-environmental quality. This differs from the findings of Sha, Chen [66] and Sun, Han [67], who state that the Chinese government should adhere to the polycentric space strategy to improve environmental quality.

### 4.2. Robustness check

#### 4.2.1. Variable substitution

Lnleeq2 is used as a substitute variable of lnleeq. In this paper, the principal component analysis method is used to calculate the indicators in Table 1 and recalculate the urban eco-environmental quality. Columns (1)-(3) in Table 3 show the estimated effect of lnleeq2 as the dependent variable. No control variables are added in columns (1) and (2), and all control variables are added in column (3), and bidirectional fixed effect model is used for estimation. The results show that at the significance level of 1%, HSR construction can significantly improve urban eco-environmental quality, and there is still a U-shaped relationship between urban space expansion and the eco-environmental quality. Therefore, the main findings are robust after substituting the dependent variable.

**Table 3:** Robustness checks.

Variable	Variable substitution			Exclude special samples			PSM-DID (noreplacement)		
	Lnleeq2			lnleeq			lnleeq		
	(1)	(2)	(3)	(4)	(5)	(6)	(7)	(8)	(9)
**HSR**_**it**_	0.053*** (0.003)	0.036*** (0.031)	0.011*** (0.003)	-0.071*** (0.022)	0.035*** (0.010)	0.029*** (0.009)	0.013** (0.006)	0.009* (0.005)	0.017** (0.004)
**lnpgdp**		-0.430*** (0.044)	-0.258** (0.039)		-6.336** (0.465)	-6.246*** (0.483)		-6.614*** (0.411)	-6.735*** (0.431)
**sqlnpgdp**		0.022*** (0.002)	0.014*** (0.002)		0.285*** (0.023)	0.279*** (0.023)		0.299*** (0.020)	0.303*** (0.020)
**US**		0.294*** (0.029)	0.381*** (0.032)		2.209*** (0.420)	1.680*** (0.324)		4.528*** (0.617)	2.907*** (0.502)
**US** ^**2**^		-0.093** (0.013)	-0.114*** (0.014)		-0.562*** (0.144)	-0.503*** (0.107)		-1.799*** (0.260)	-1.445*** (0.208)
**HC**			0.013 (0.003)			0.026** (0.010)			0.031*** (0.015)
**FDI**			-0.176 (0.112)			-0.027 (0.187)			0.015 (0.187)
**INS**			-0.200** (0.085)			-0.974** (0.417)			-1.238*** (0.457)
**RD**			0.021*** (0.002)			0.025*** (0.009)			0.019* (0.010)
**N**	4,913	4,663	4,259	3,800	3,708	3,473	2,423	2,364	2,387
**Adj -R** ^**2**^	0.779	0.962	0.969	0.818	0.965	0.971	0.854	0.932	0.945

Note

Inside the bracket is the standard error of cluster and city

*p<0.10, **p<0.05, ***p<0.01.

#### 4.2.2. Special samples are excluded

China’s carbon emissions trading pilot policy will affect environmental performance [68], therefore, it is necessary to exclude the role of carbon emissions trading system on the eco-environmental quality. Table A2 in S1 File shows the timeline for the implementation of carbon emission trading pilot policies in parts of China. Samples that participated in the pilot carbon trading policy during the sample period were removed and the estimates obtained are shown in columns (4)-(6) of Table 3. After the control variables are added, the estimated coefficient of HSR opening term is 0.029, which is significant at 1% level. The estimated coefficient of urban space expansion is 1.680, and the estimated coefficient of its square term is -0.503, which is significant at the level of 1%. The magnitude, direction and significance levels of the estimated coefficients are similar to the basic estimates, further validating the robustness of our conclusions.

#### 4.2.3. The PSM-DID estimate

In this paper, cities with HSR service from 2008 to 2020 were selected as the experimental group, and other cities were selected as the control group. The method of phase-by-phase matching is adopted [69, 70]. The method is based on the principle of nearest neighbor matching without substitution to determine the weight of the city in each year according to the ratio of one to one. The propensity score was estimated using the logit model, and then the samples were pooled together, and the multi-time DID estimation was performed for the matched treatment and control groups according to Formula (1).

**Fig 2:**
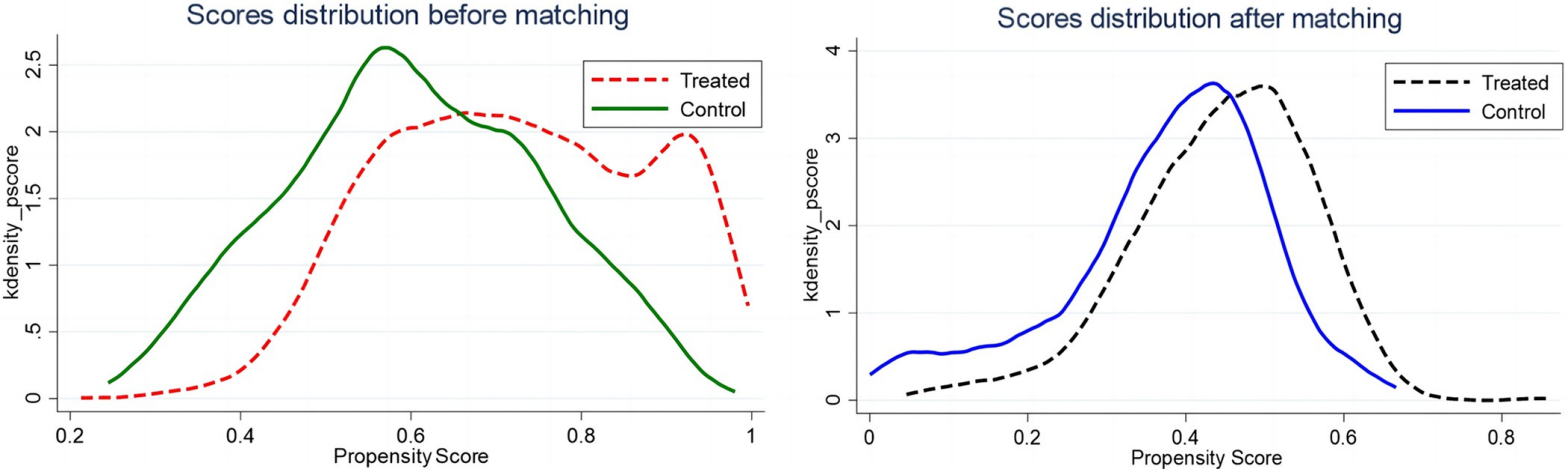
Kernel density function curves for the treatment and control groups.

Kernel density function curves were plotted for treatment and control groups after nearest neighbor propensity score matching (Fig 2). In Fig 2, compared with before matching, the probability density distributions of the two groups after matching tend to be significantly consistent, indicating that the characteristics of the two groups are closer after matching, which eliminates the selectivity bias of the samples. The balance test results are shown in Table 4. The results showed that the T-test of all matched variables in the matched samples was not significant, indicating that there was no mean difference between groups in the matched variables, and the standardized differences of the matched variables all decreased significantly after matching. From the value of Pseudo R^2^, it appears that R^2^ is small after matching. The results of propensity score matching are reported in Fig A1 in S1 File. It means that the matching variable has a weak explanatory power for whether a city has opened HSR, that is, whether the city has opened HSR is random to the matched sample. The estimation results of PSM-DID are reported in columns (7)-(9) of Table 3. The results show that the coefficients of main variables are consistent with the baseline estimates at the 10% level of significance, implying that our findings are robust.

**Table 4:** The balance test results.

Variables	Matching? (Y/N)	Mean			Standardized Difference Test	
		Treated mean	Control mean	t-test (P-Value)	%bias	% reduct |bias|
**US**	NO	0.233	0.276	-5.80 (0.000)	-27.7	83.7
	YES	0.238	0.231	0.86 (0.389)	4.5	
**US** ^**2**^	NO	0.072	0.106	-5.64 (0.000)	-27.5	84.6
	YES	0.076	0.071	0.89 (0.375)	4.2	
**lnpgdp**	NO	10.720	10.519	6.07 (0.000)	34.1	84.4
	YES	10.496	10.528	-0.86 (0.390)	-5.3	
**sqlnpgdp**	NO	115.290	110.99	6.09 (0.000)	34.3	85.8
	YES	110.550	111.16	-0.79 (0.428)	-4.9	
**HC**	NO	2.176	2.173	0.72 (0.47)	4.1	88.4
	YES	2.176	2.176	0.08 (0.937)	0.5	
**FDI**	NO	0.577	0.636	-1.31 (0.191)	-7.5	64.5
	YES	0.577	0.598	-0.46 (0.644)	-2.6	
**INS**	NO	16.635	16.853	-1.31 (0.191)	-24.3	89.1
	YES	16.637	16.661	-0.45 (0.650)	-2.6	
**RD**	NO	0.859	1.108	-5.39 (0.000)	-31.6	91.3
	YES	0.861	0.882	-0.61 (0.541)	-2.8	
**Pseudo R** ^**2**^	NO	0.058				
	YES	0.005				

### 4.3. Endogeneity and the instrumental variable approach

There may be omitted variables in the above estimation model, such as institutional factors and urban resource endowment. Therefore, this paper further employs an instrumental variables approach based on the baseline regression to correct for the estimation bias caused by the potential endogeneity problem of the model. For the selection of instrumental variables, this paper draws on the idea of selecting geographic characteristics and sample exclusion methods [71, 72], using slope gradient and eliminating central cities (municipalities and provincial capitals) to alleviate the endogeneity problem. First, the important goal of HSR construction is to shorten the spatio-temporal relationship between cities and improve inter-city accessibility. Central cities, mainly including municipalities directly under the central government and provincial capitals, with large population size and economic volume, can easily become node cities, and removing such cities can alleviate endogenous problems. Second, the slope gradient affects the opening cost of HSR to a certain extent, which can meet exogenous conditions. In this paper, the interaction between slope gradient and dummy variables is added to the benchmark model to avoid multicollinearity.

The results of instrumental variable method are shown in Table 5, where (1) is the estimation result including all samples, and (2) is the estimation result after excluding central cities. The first-stage estimation results all show that the instrumental variables are significantly negative, and the goodness of fit is more than 70%, so it has a good interpretation of urban eco-environmental quality. In the two-stage regression of the instrumental method, the estimated coefficients of the HSR opening were significantly positive, compared with the baseline estimate of 0.028 (second column in Table 2), and the estimated coefficients of the HSR opening in (1) and (2) were 0.038 and 0.050, respectively, meaning that the impact of HSR opening on eco-environmental quality was significantly higher. Thus, after eliminating the potential endogenous problems, it can be seen that the impact of HSR opening on the eco-environmental quality of cities is more prominent.

**Table 5:** Regression results of instrumental variables.

Variables	(1)	(2)
**HSR**	0.038*** (0.007)	0.050*** (0.008)
**lnpgdp**	-6.426*** (0.151)	-6.613*** (0.163)
**sqlnpgdp**	0.287*** (0.007)	0.296*** (0.008)
**US**	0.288*** (0.029)	0.340*** (0.040)
**US** ^**2**^	-0.093*** (0.013)	-0.132*** (0.021)
**Other control variables**	YES	YES
**City fixed effect**	YES	YES
**Year fixed effect**	YES	YES
**N**	4,343	3,897
**R** ^**2**^	0.970	0.970
First-stage regressions		
**ln_tor**	-0.240*** (0.006)	-0.242*** (0.007)
**adj-R** ^**2**^	0.737	0.734
**F statistic**	98.700	91.060
**N**	4,343	3,897

Note

Inside the bracket is the standard error of cluster and city

*p<0.10

**p<0.05

***p<0.01.

## 5. Heterogeneity analysis

### 5.1. Heterogeneity analysis based on the characteristics of HSR connected cities

Next, we estimate models (2), (3), and (4) to examine the effects of interactions between cities with different levels of economic development, industrial structures, and population sizes via HSR connectivity on eco-environmental quality.

Table 6 reports the estimated results. Among them, columns (1) and (2) show the effect of HSR connectivity between cities with different economic development levels on eco-environmental quality. Columns (3) and (4) show the effect of HSR connectivity between cities with different industrial structures on eco-environmental quality. Columns (5) and (6) show the effect of HSR connectivity between cities with different population sizes on eco-environmental quality. The results show that HSR connectivity with cities with higher economic development levels, industrial structure and population size has a positive effect on the eco-environmental quality. It means that large cities, mainly developed cities, do not pay enough attention to eco-environmental quality in the process of improving infrastructure construction and developing the economy. The possible reasons are as follows: On the one hand, due to the labor mobility bias, in reality, labor mobility occurs more frequently between cities with large differences in economic development level and economic structure to achieve spatial arbitrage, and the increasing floating population in cities with higher economic development level leads to the increasing ecological pressure. On the other hand, because in the period of industrial restructuring, the rapid transformation from the secondary industry to the tertiary industry may weaken the “structural dividend” of traditional economic development, which is not conducive to economic growth and is contrary to the economic development goals of local governments. Therefore, the governments of some service-oriented cities still pay more attention to the secondary industry development, and the manufacturing industry is still expanding while developing the tertiary industry. According to the study of Chang, Diao [4], HSR operation leads to the decentralization and agglomeration of manufacturing and service industries, respectively. It is also not conducive to improving the eco-environmental quality of service-oriented cities if the HSR connection leads to the agglomeration of service industries from manufacturing-oriented cities to service-oriented cities.

**Table 6:** Estimation results of HSR connectivity between different cities.

Variables	(1)	(2)	(3)	(4)	(5)	(6)
**HSR**_**it**_	0.074** (0.029)	0.030*** (0.026)	0.067** (0.026)	0.030*** (0.026)	0.030*** (0.009)	0.024*** (0.008)
**HSR_hpgdp**	0.101** (0.047)	0.056*** (0.016)				
**HSR_lpgdp**	-0.040 (0.059)	0.015 (0.017)				
**HSR_lis**			-0.054** (0.022)	-0.007 (0.010)		
**HSR_his**			0.081*** (0.030)	0.030** (0.012)		
**HSR_lpop**					-0.027 (0.030)	-0.005 (0.021)
**HSR_hpop**					0.043** (0.017)	0.033** (0.014)
**Control variables**	NO	YES	NO	YES	NO	YES
**City fixed effect**	NO	YES	NO	YES	NO	YES
**Year fixed effect**	NO	YES	NO	YES	NO	YES
**N**	4,760	4,343	4,760	4,343	4,760	4,343
**Adj-R** ^**2**^	0.780	0.838	0.778	0.968	0.777	0.968

Note

Inside the bracket is the standard error of cluster and city

*p<0.10, **p<0.05, ***p<0.01.

### 5.2. Heterogeneity analysis based on urban agglomeration

**Fig 3:**
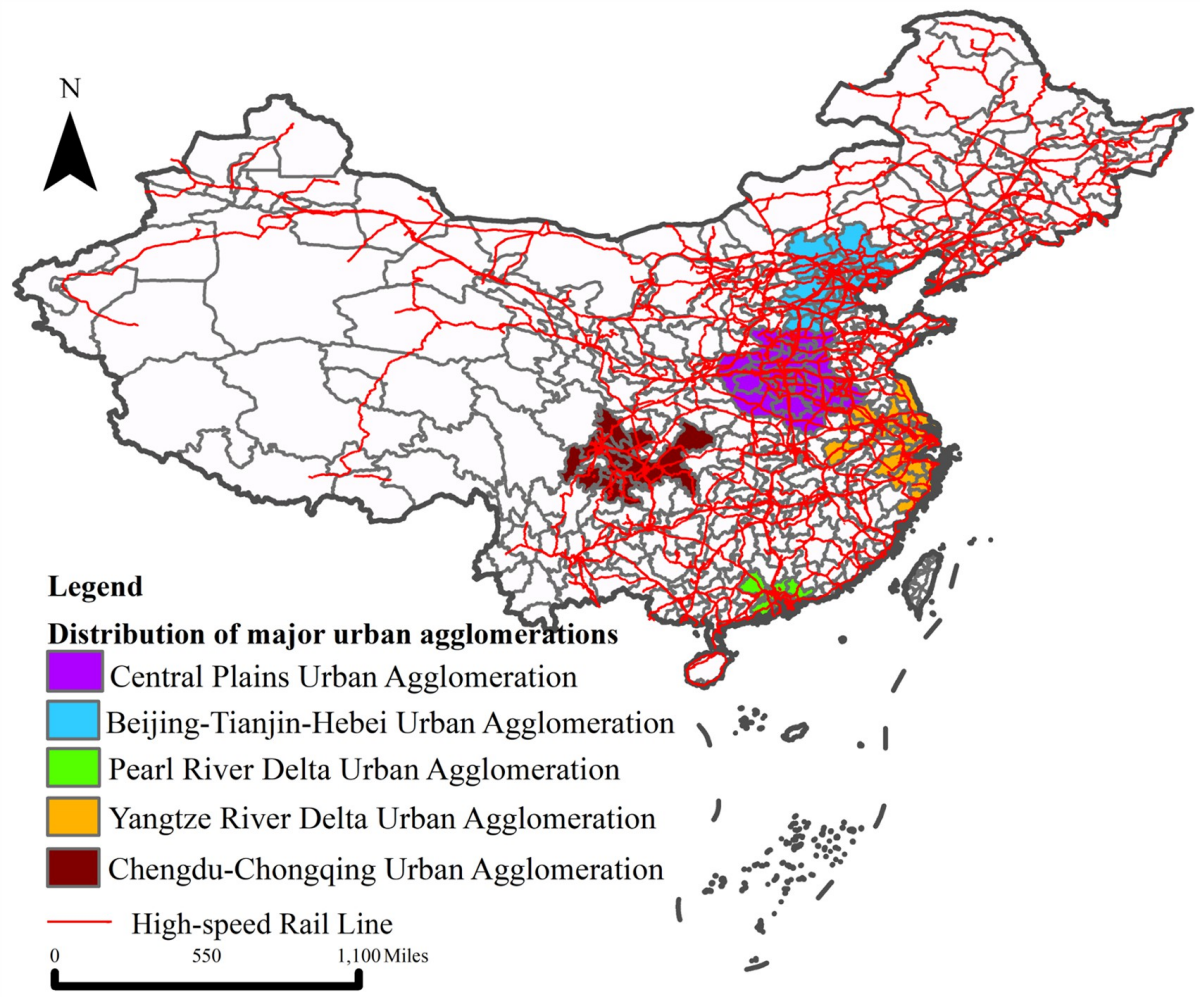
HSR network of China, 2019. Note: Based on the standard map of the Map Technical Review Center of the Ministry of Natural Resources of China (Review No. GS(2020)4619), with no modifications to the boundaries of the base map.

Urban agglomeration is an important carrier to promote coordinated regional development and an important platform to support economic development. In 2020, China’s 19 urban agglomerations gather 75% of the population on 25% of the land and generate 88% of the GDP. At present, China has formed a HSR distribution pattern of “four-vertical-and-four-horizontal”, and a number of obvious regional group sub networks have appeared in the HSR network, which is highly consistent with the distribution of urban agglomeration. Considering the large differences in population, economy and regional prosperity among urban agglomerations, and the effect of HSR on the eco-environmental quality among urban agglomerations will be affected by urban agglomeration effect, diffusion effect, multiplier effect and other effects [40], we further analyzed the heterogeneity from the perspective of urban agglomerations. Fig 3 is the distribution map of China’s HSR lines in 2019. It can be found that the HSR lines within urban agglomerations are relatively well established. This paper analyses five urban agglomerations in China that are economically mature, dynamic and have a wide coverage. These five urban agglomerations have good performance in economic strength, population size and online popularity, and are the main representatives of Urban agglomerations in China.

Table 7 shows the estimated results of the impact of HSR construction on eco-environmental quality in different urban agglomerations. The results show that HSRs construction have significant positive effects on eco-environmental quality of the Central Plains urban agglomeration (CPUA), the Beijing-Tianjin-Hebei urban agglomeration (BTHUA), the Pearl River Delta urban agglomeration (PRDUA) and the Yangtze River Delta urban agglomeration (YRDUA), the Yangtze River Delta urban agglomeration and the Chengdu-Chongqing urban agglomeration (CCUA). Among them, the YRDUA has the strongest positive effect on eco-environmental quality, and the CPUA has the weakest effect. The reasons lie in the relatively weak economic strength of CPUA, the integration between cities in the region to be improved, the high dependence of economic growth on resource and energy consumption, and the insufficiently strong driving effect of infrastructure upgrading on clean industries, which is reflected in the fact that after the HSR opening, most of the industries transferred to the region are low value-added and low-technology capital- and labour-intensive industries, resulting in the weak influence of the improvement of transportation conditions on environmental pollution. In comparison, the YRDUA is one of the most mature urban agglomerations in China. Cities have different industrial structures but similar economic development levels. HSR opening is conducive to the division of work and cooperation between cities using their comparative advantages to achieve dislocation development, improve production efficiency, and ultimately improve the eco-environmental quality. The transportation network within the YRDUA is denser, the services that accompany it are more complete [73], and the exchange and distribution of resource elements generated by the transportation network are more frequent, which is conducive to the diffusion and application of cleaner technologies, and makes a more significant marginal contribution to the emissions of environmental pollution.

**Table 7:** Heterogeneity test results based on urban agglomeration.

Variable	CPUA		BTHUA		YRDUA		PRDUA and YRDUA		CCUA	
**HSRit**	0.016* (0.009)	0.007* (0.004)	0.086*** (0.021)	0.023*** (0.008)	0.060*** (0.018)	0.044*** (0.016)	0.034** (0.015)	0.029*** (0.002)	0.051*** (0.022)	0.012*** (0.005)
**lngdp**		-5.856*** (0.357)		-3.498*** (0.341)		-3.638*** (0.079)		-3.543*** (0.063)		-7.369*** (0.487)
**sqlngdp**		0.264*** (0.018)		0.153*** (0.016)		0.156*** (0.004)		0.152*** (0.003)		0.337*** (0.025)
**US**		0.479 (0.841)		0.634*** (0.237)		0.708*** (0.187)		2.127*** (0.297)		0.575*** (0.237)
**US** ^**2**^		-1.160* (0.603)		-0.320 (0.288)		-1.347*** (0.499)		-5.814*** (0.879)		-0.988** (0.283)
**N**	416	416	240	240	391	391	527	496	254	239
**R2**	0.740	0.680	0.887	0.987	0.788	0.975	0.777	0.974	0.779	0.979
**Adj-R** ^**2**^	0.713	0.643	0.871	0.985	0.764	0.972	0.755	0.972	0.772	0.975

Note

Inside the bracket is the standard error of cluster and city

*p<0.10, **p<0.05, ***p<0.01.

According to the results, there is a U-shaped relationship between urban spatial expansion (US) and eco-environmental quality in the YRDUA, the PRDUA and YRDUA, and the CCUA, and about 20% of the cities have caused damage to the eco-environmental quality due to excessive expansion. There is a significant positive correlation between spatial expansion and eco-environmental quality in the BTHUA. The reason is that 50% of the population in the Beijing Tianjin Hebei urban agglomeration is distributed in Beijing, Tianjin, Shijiazhuang and Baoding, and the total amount and quality of economic development among cities are not coordinated. However, since 2015, Beijing has made good progress in promoting population dispersion by function and industry dispersion. The cooperation of urban agglomeration in the three key areas of transportation, ecology and industry has made remarkable achievements. On the whole, the BTHUA promotes the urban land-conversion process efficiently by virtue of the perfect transportation infrastructure and the construction of the urban spatial pattern of Beijing with “one body and two wings”. Meanwhile, it also gives play to the radiation and spillover effects of Beijing’s innovative resources to improve the eco-environmental quality in all aspects.

## 6. Conclusion and discussion

At present, China has entered the “HSR era”, the HSR network has an important impact on regional economic development and ecological environment. Based on China’s urban panel data, this paper empirically examines the impact of HSR construction on eco-environmental quality and its heterogeneity. First, we found that HSR has significantly improved the eco-environmental quality. Compared with cities without HSR service, the eco-environmental quality of cities with HSR service has significantly increased by 0.023 standard deviations, which is about 4.11% of the total change of urban eco-environmental quality in the same period. Second, there is an inverted U-shaped relationship between eco-environmental quality and urban space expansion. Finally, the impact of HSR opening in different cities and urban agglomerations on eco-environmental quality is heterogeneous. Relevant policy suggestions are as follows:

First, as an environment-friendly intercity transportation tool, China’s high-speed rail is an important infrastructure to change the urban spatial and geographical pattern. The HSR network plays a positive role in improving eco-environmental quality. Therefore, in the long run, the government should give full play to the advantages of transport infrastructure development in terms of population mobility and resource integration, strengthen the interface between the HSR and the urban public transport system, and maximize the green attributes of the HSR. Second, if cities with low economic development levels, industrial structure and population size are connected with cities with high economic development levels, high industrial structure and large population sizes through HSR, the HSR opening will have a more significant positive impact on their eco-environmental quality. Depending on the level of industrial development and the level of infrastructure development in the different regions, it is, therefore, necessary to have reasonable guidance, a scientific formulation of industrial development and planning of industrial transfer. For example, in creating a regional economic alliance, the government should encourage the development of differentiated high-tech industries in developed regions, optimize the business environment, and give full play to the positive effect of infrastructure upgrades on resource allocation efficiency, thereby promoting green transformation of firms and fundamentally supporting the green development of the economy. Moreover, the effect of urban economic growth on the eco-environmental quality in China has not yet reached the inflection point. This means that for most cities in China, population and economic growth are still important sources of ecological pressure. Thus, the government also needs to implement strict environmental regulation policies to strengthen ecological and environmental conservation. Third, in the process of achieving the goal of high-quality development of urban agglomerations based on modern transportation networks, the government should reasonably arrange the construction land, adopt a reduction policy to promote the transformation of land development to intensive and efficient, to reduce the negative impact on eco-environmental quality in the process of production and living.

This study, however, has some limitations. The networked layout of HSR has changed the location advantage of cities in the entire regional network, and the impact of transportation infrastructure on the ecological environment is becoming more and more networked. In addition, research on a single scale or one mode of transportation is difficult to depict the overall picture of spatial correlation between cities, so it is necessary to compare and analyze the characteristics of aviation and HSR networks. In the future, we will consider combining the complex network analysis method with the econometric research method to further study the environmental impact of infrastructure construction from the network characteristics of rapid transit.

## Supporting information

S1 File (DOCX)

S1 Data (XLSX)
