# Supplementary material for: Transportation infrastructure and eco-environmental quality: Evidence from China’s high-speed rail
Source: PLoS One. 2023 Aug 29;18(8):e0290840. doi: 10.1371/journal.pone.0290840 (PMC10465004; doi:10.1371/journal.pone.0290840)
Supplement: S1 File — (DOCX) [file pone.0290840.s001.docx]

**Appendix**

**
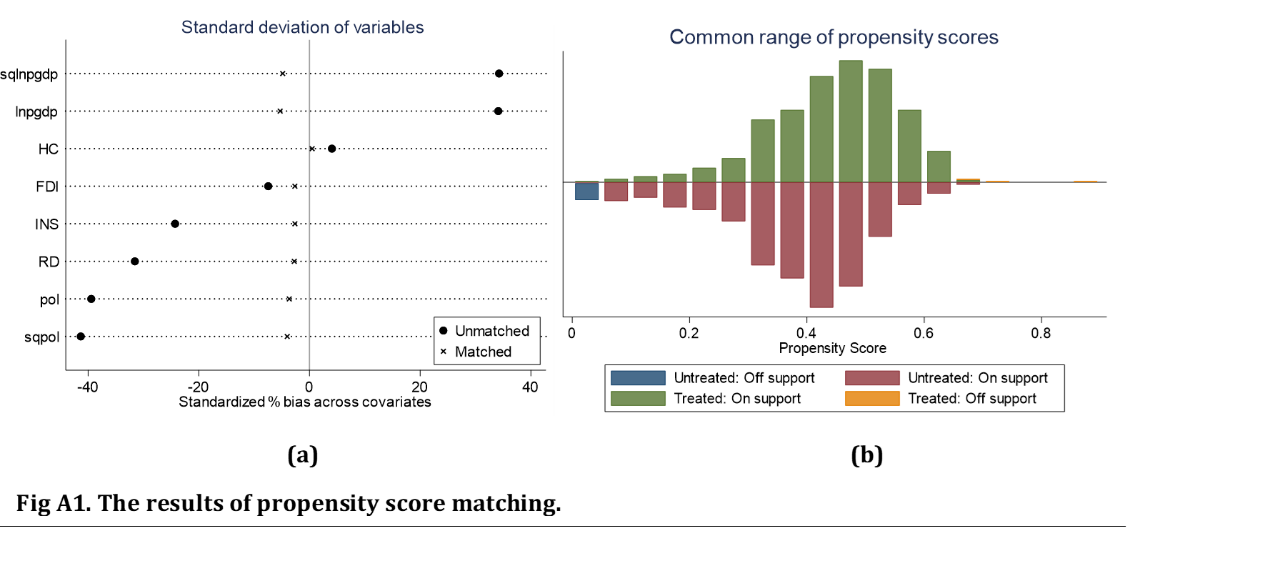
**

**Table A1. Descriptive statistics of variables.**

| **variable** | **Total sample** | | | **Control group** | | | **Treatment group** | | |
| --- | --- | --- | --- | --- | --- | --- | --- | --- | --- |
|  | Obs. | Mean | Std. Dev. | Obs. | Mean | Std. Dev. | Obs. | Mean | Std. Dev. |
| **Leeq1** | 4,913 | 0.366 | 0.066 | 1,203 | 0.385 | 0.067 | 3,710 | 0.360 | 0.065 |
| **Leeq2** | 4,913 | 1.084 | 0.171 | 1,203 | 1.134 | 0.179 | 3,710 | 1.068 | 0.168 |
| **HSR_it_** | 4,913 | 0.316 | 0.465 | 1,237 | 0.000 | 0.000 | 3,727 | 0.421 | 0.494 |
| **US** | 4,525 | 0.366 | 0.183 | 1,054 | 0.572 | 0.504 | 3,471 | 0.304 | 0.308 |
| **lnpgdp** | 4,524 | 5.415 | 1.142 | 1,053 | 5.340 | 1.061 | 3,471 | 5.438 | 0.494 |
| **FDI** | 4,406 | 0.019 | 0.018 | 984 | 0.013 | 0.016 | 3,422 | 0.021 | 0.016 |
| **INS** | 4,807 | 47.085 | 11.293 | 1,119 | 46.932 | 14.183 | 3,688 | 47.132 | 10.258 |
| **HC** | 4,701 | 4.319 | 1.316 | 1,032 | 3.641 | 1.258 | 3,669 | 4.509 | 1.268 |
| **RD** | 4,524 | 3.685 | 1.575 | 1,053 | 3.512 | 1.438 | 3,471 | 3.738 | 1.603 |
| **lnfrequencies_it_** | 2,928 | 1.473 | 2.061 | 1014 | 0.000 | 0.000 | 2,191 | 1.905 | 2.171 |
| **Routes_it_** | 4,913 | 0.284 | 0.806 | 737 | 0.000 | 0.000 | 3,727 | 0.374 | 0.221 |

**Table A2. Carbon Emissions Pilot Policy Trading Schedule by Region in China.**

| **Area Name** | **Timing of carbon trading market establishment** | **Area Name** | **Timing of carbon trading market establishment** |
| --- | --- | --- | --- |
| **Beijing** | 28 November 2013 | **Chongqing** | 19 June 2014 |
| **Tianjin** | 26 December 2013 | **Guangdong Province (except Shenzhen)** | 19 December 2013 |
| **Hubei Province** | 12 April 2014 | **Shenzhen** | 18 June 2013 |
| **Shanghai** | 26 November 2013 |  |  |
